# Detection of circulating tumor DNA in patients with advanced non-small cell lung cancer

**DOI:** 10.18632/oncotarget.12883

**Published:** 2016-10-25

**Authors:** Yu Yao, Jinghao Liu, Lei Li, Yuan Yuan, Kejun Nan, Xin Wu, Zhenyu Zhang, Yi Wu, Xin Li, Jiaqi Zhu, Xuehong Meng, Longgang Wei, Jun Chen, Zhi Jiang

**Affiliations:** ^1^ Department of Medical Oncology, The First Affiliated Hospital of Xi’an Jiaotong University, Shanxi, China; ^2^ Department of Lung Cancer Surgery, Tianjin Key Laboratory of Lung Cancer Metastasis and Tumor Microenvironment, Tianjin Lung Cancer Institute, Tianjin Medical University General Hospital, Tianjin, China; ^3^ Novogene Bioinformatics Institute, Beijing, China

**Keywords:** circulating tumor DNA, NSCLC, targeted sequencing, EGFR, gene fusion

## Abstract

Circulating tumor DNA (ctDNA) isolated from plasma has great potential in identification of gene mutation in non-small cell lung cancers (NSCLC), which is a non-invasive technique and can avoid the inherent shortcomings of tissue biopsy. However the ability of NGS to detect gene mutation in plasma ctDNA has not been broadly explored. To assess the diagnostic ability of ctDNA for the total mutation profile, including single nucleotide variations (SNVs), insertions and deletions (indels) and gene rearrangements, we performed a targeted DNA sequencing approach to screen NSCLC related driver gene mutations in both tissue biopsies and matched blood plasma samples from 39 advanced NSCLC patients from China. The sensitivity of *EGFR*, *KRAS*, *PIK3CA* mutations and gene rearrangements detected in plasma ctDNA was 70.6%, 75%, 50% and 60%, respectively and the overall concordance of gene mutations between tissue DNA and plasma ctDNA was 78.21%. Our data provide evidence that ctDNA in plasma is likely to become an alternative source for cancer-related mutations profiling in advanced NSCLC patients and targeted sequencing of ctDNA offers a promising perspective on precise diagnostics and may serve as a feasible option for clinical monitoring of NSCLC patients.

## INTRODUCTION

Lung cancer is the leading cause of cancer-related mortality in China [1]. Non-small cell lung cancer (NSCLC), the most common subtype of lung cancer, is often characterized by unique driver gene mutation profiles [2–4]. Precise characterization of tumor mutation profiles is a fundamental part of personalized therapy. Tissue biopsies obtained in surgery are the “gold standard” for detecting oncogene mutations [5]. However, tissue biopsies have some inherent shortcomings in clinical practice. Mutations detected in different metastatic clones can be significantly diverse from each other or from the primary tumor tissue [6]. ctDNA is generated from apoptotic or necrotic tumor cells, circulating tumor cells or metastatic tumors. DNA fragments carrying tumor-specific genetic alterations can be extracted from blood plasma for further examination [7–9]. This non-invasive type of “liquid biopsy” can be taken easily and repeatedly over the course of a patient's treatment, and meanwhile, ctDNA may potentially reflect all heterogeneous genetic mutation profiles, with overcoming other common obstacles in conventional tissue biopsy. Given that, ctDNA provides new insight into diagnosis, prognosis and patient follow-up compared to traditional tissue biopsy.

Somatic mutation analysis of known oncogenes, such as epidermal growth factor receptor (*EGFR*), has become a routine clinical test in NSCLC for patient prognosis and targeted drug selection [10]. Approximately 10% of patients with NSCLC in the US and 35% in East Asia have somatic *EGFR* mutations [11]. *EGFR* mutation status is an useful predictor of efficacy for *EGFR* tyrosine kinase inhibitors (EGFR-TKIs) [12]. Over the past decade, there is increasing evidence that *ALK* rearrangements are more commonly found in NSCLC patients who are light smokers or never smokers. And *ALK* rearrangements are also associated with younger age and adenocarcinomas. Most recently, EML4-*ALK* fusions are recognized to be potential driver mutations in NSCLC. NSCLC patients harboring *ALK* fusions derive more benefits from ALK-TKIs. Therefore, from a clinical perspective, it is essential to accurately and comprehensively assess tumor-related gene mutation profiles, including SNVs, indels and gene rearrangements in NSCLC patients.

In recent years, several studies have confirmed that NSCLC-related driver gene mutations (such as *EGFR* and *KRAS*) could be detected in plasma DNA by a variety of methods, including BEAMing (beads, emulsion, amplification, and magnetics) technology [13], peptide nucleic acid (PNA)-mediated polymerase chain reaction (PCR) [14] and the Scorpion ARMS-based *EGFR* mutation detection method [15]. And it is reported that *EGFR* exon 19 deletion and L858R mutation would be detected from circulating cell-free DNA from NSCLC patients [16]. Concordant NSCLC driver gene profiles between ctDNA and primary tumor DNA has been reported by several groups. Using mutant enriched liquid chip (MEL), Zhang has detected *EGFR, KRAS, BRAF* and *PIK3CA* in 86 tissue samples and matched plasma samples in NSCLC patients, with overall agreements of 64%, 97%, 98% and 97%, respectively [17]. NSCLC driver gene mutations in matched tumor DNA and ctDNA have also been identified by the semiconductor-based targeted sequencing method, with an overall concordance of 76% [18]. Using Ion Torrent's Ampliseq hotspot cancer panel, Ronald Lebofsky and his colleagues demonstrate that 28 of 29 mutations detected in metastasis biopsies have also been found in matched ctDNA among 27 samples [19]. However, previous studies are focused on SNVs and indels of the driver genes, whether the gene rearrangement detected from plasma ctDNA is accordance with that in tissue DNA has not been illustrated yet. Fusions of oncogenes, such as *ALK*, *ROS1* and *RET*, are involved tumorigenesis and are recognized as likely future predictive lung tumor biomarkers [20]. Detection of gene rearrangements from plasma ctDNA would have a substantial impact on the clinical diagnostic and prognostic stratification of NSCLC.

In this study, we used a targeted sequencing approach based on the Illumina platform to detect and compare NSCLC driver gene alterations, including point mutations, indels and gene rearrangements, simultaneously in tissue biopsies and matched plasma samples from 39 Chinese patients with advanced NSCLC.

## RESULTS

### Patient characteristics

Tissue and matched peripheral blood samples obtained from 39 NSCLC patients were analyzed. Patient clinical characteristics are listed in Table 1. Participants in this study cohort, including 20 females and 19 males, were diagnosed with stage IIIa to IV NSCLC. A total of 34 of 39 patients (87%) had adenocarcinoma and 5 of 39 patients (13%) had squamous cell carcinoma. The majority of patients (29/39, 74.4%) were non-smokers. Most of patients (36 of 39, 92.3%) were treatment naïve. In the three treatment-experienced patients, two had first line target therapy during the past 1.5 years and the other one received chemotherapy.

**Table 1:** Clinical features of 39 patients with non-small cell lung cancer

Characteristics	Number
Age (years)	
Mean (SD)	59 (11.60)
Median (range)	62 (28–78)
Sex	
Male	19 (48.7%)
Female	20 (51.3%)
Pathological diagnosis	
Non-small cell lung cancer	39 (100.0%)
Adenocarcinoma	34 (87.2%)
Squamous cell carcinoma	5 (12.8%)
Tumor stage	
IIIA	5 (12.8%)
IIIB	3 (7.7%)
IV	31 (79.5%)
Smoking history	
Smoker	10 (25.6%)
Non-smoker	29 (74.4%)
Treatment history	
Treatment naïve patients	35 (89.7%)
Treatment experienced patients	4 (10.3%)
Chemotherapy history	
Undertook chemotherapy	1 (2.6%)
Not undertook chemotherapy	38 (97.4%)
Targeted therapy history	
Treatment with target therapy	1 (2.6%)
Not treatment with target therapy	38 (97.4%))

### Sequencing coverage analysis

**Figure 1:**
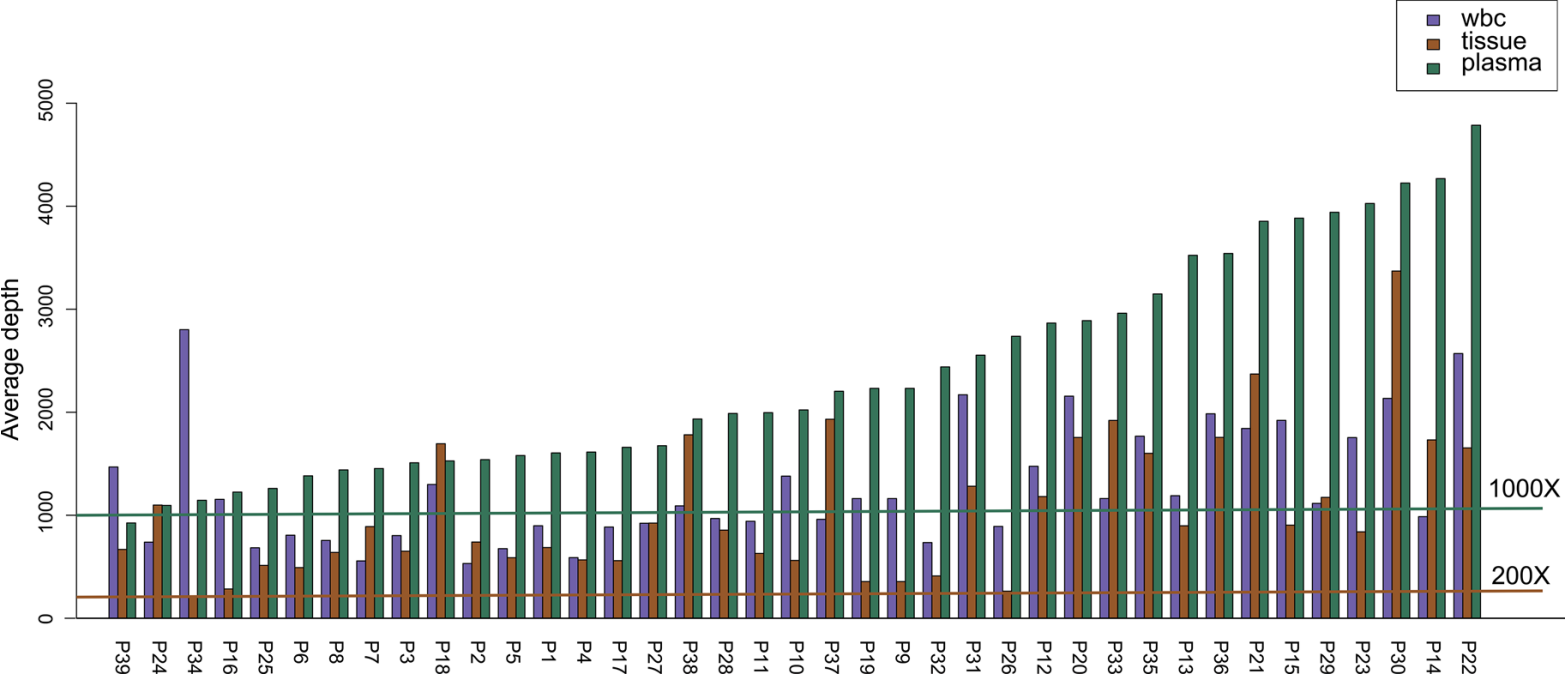
Average depth of the NGS assay Average sequencing depth of target in 39 matched tissue, plasma and white blood cell (WBC) samples. For the tissue and WBC samples, average sequencing depth of target is more than 200× (brown line). For plasma samples, average sequencing depth of target is more than 1000× (dark green line).

All samples, consisting of plasma, white blood cell (WBC) and tissue, were sequenced using paired-end strategy on an Illumina HiSeq platform. More than 90% of the bases had a phred quality score greater than 20 (error rate less than 1%). We achieved an average sequencing depth on target in all tested samples: median depth of 855.17× (range 215.57×–3370.70×) for tissue DNA samples, median depth of 2022.29× (range 925.00×–4786.10×) for plasma ctDNA samples, and median depth of 1114.52× (range 531.29×–2801.77×) for WBC samples (Figure 1).

### Comparison of matched tissue and plasma mutations

**Figure 2:**
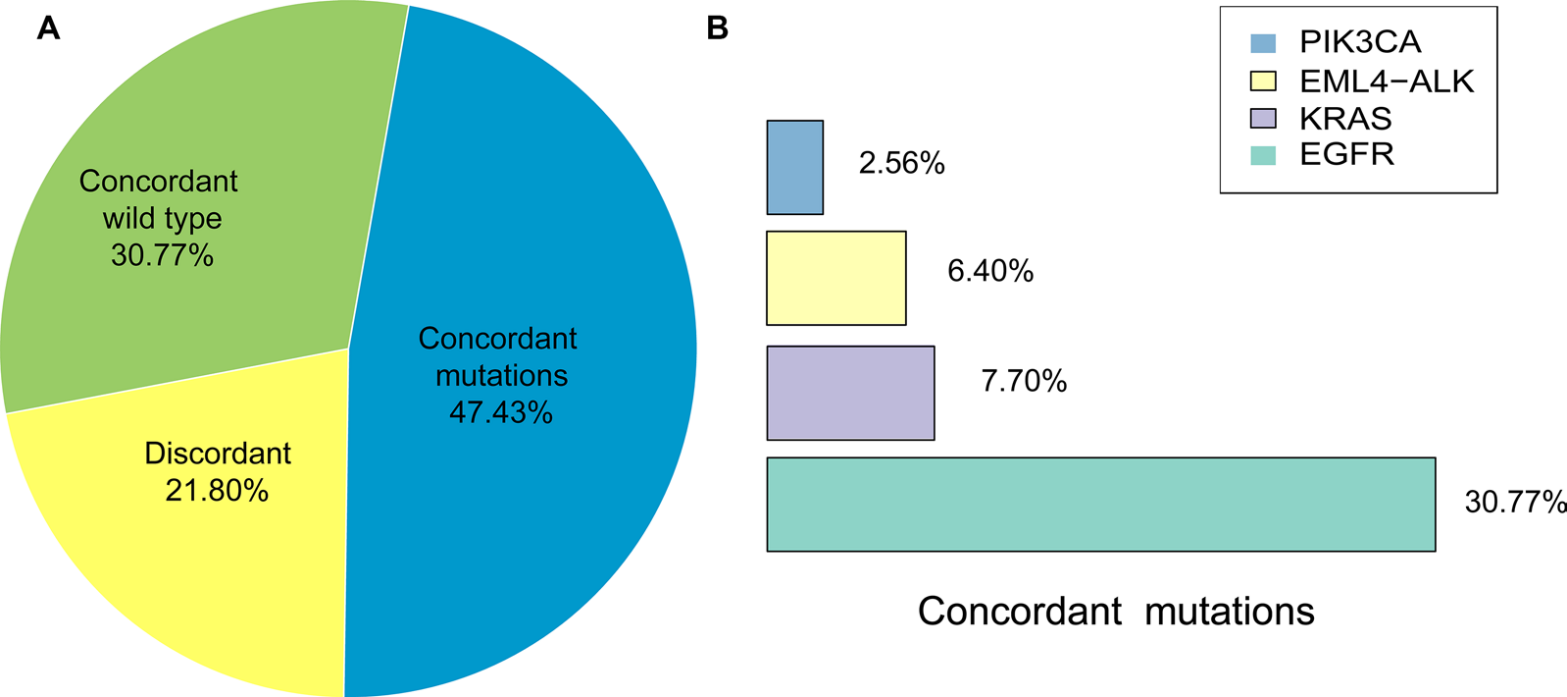
Comparison of matched tissue and plasma mutations (**A**) Percentage of concordant (wild type and mutations) and discordant mutations in matched tissue and plasma samples. (**B**) Percentage of NSCLC-related driver gene mutations detected in both matched tissue and plasma samples.

SNVs, Indels and fusions of NSCLC-related genes, including *EGFR*, *KRAS*, *PIK3CA*, *ALK* and *RET*, detected in 39 advanced NSCLC patients were listed in Tables 2 and 3. The concordant mutations detected both in tissue DNA and plasma ctDNA in 18.5 of 39 patients (47.43%), and no somatic mutations were found from both samples in 12 patients (30.77%), and mutations were only found from tissue DNA in 8.5 patients (21.80%) (Figure 2A). Gene mutations detected in both tissue DNA and plasma ctDNA were *EGFR*, *KRAS*, *ELM4-ALK* rearrangements and *PIK3CA*, and the overlap mutation rates in tissue and plasma of them were 30.77%, 7.70%, 6.40% and 2.56%, respectively (Figure 2B).

**Table 2:** Single nucleotide variations and indels detected in tissue DNA and plasma ctDNA

Patients	Cancer type	Tumor stage	Position	Gene	Mutation	Mutation type	% variants			Concordance (Yes or No)
							Tissue DNA	Plasma DNA	WBC DNA	
P4	AC	IIIB	chr7:55249071	*EGFR*	p.T790M	SNV	0.30	7.70	0	Yes
			chr7:55259515	*EGFR*	p.L858R	SNV	23.30	11.60	0	Yes
P6	AC	IV	chr7:55259515	*EGFR*	p.L858R	SNV	12.30	5.10	0	Yes
P8	AC	IV	chr7:55242465	*EGFR*	p.745_750del	DEL	20.00	0.20	0	Yes
P9	AC	IV	chr7:55259515	*EGFR*	p.L858R	SNV	28.50	5.00	0	Yes
P10	AC	IV	chr7:55259515	*EGFR*	p.L858R	SNV	6.00	7.00	0	Yes
P11	AC	IV	chr7:55259515	*EGFR*	p.L858R	SNV	25.00	0.70	0	Yes
P19	AC	IIIA	chr3:178936091	*PIK3CA*	p.E545K	SNV	18.70	4.90	0	Yes
P27	AC	IV	chr7:55241707	*EGFR*	p.G719S	SNV	51.00	0.20	0	Yes
P29	AC	IV	chr7:55242466	*EGFR*	p.746_750del	DEL	52.00	7.20	0	Yes
P31	AC	IV	chr7:55259515	*EGFR*	p.L858R	SNV	38.00	9.70	0	Yes
P32	AC	IV	chr7:55242466	*EGFR*	p.746_748del	DEL	34.00	9.00	0	Yes
P34	AC	IV	chr12:25398284	*KRAS*	p.G12V	SNV	28.90	9.80	0	Yes
P35	AC	IV	chr12:25398285	*KRAS*	p.G12C	SNV	19.70	5.00	0	Yes
P36	AC	IV	chr7:55242467	*EGFR*	p.746_751del	DEL	30.30	8.10	0	Yes
P37	AC	IV	chr7:55259515	*EGFR*	p.L858R	SNV	12.70	0.20	0	Yes
P39	AC	IIIB	chr12:25398284	*KRAS*	p.G12V	SNV	26.00	0.69	0	Yes
P1	AC	IV	chr3:178936091	*PIK3CA*	p.E545K	SNV	23.30	0	0	No
			chr7:55259515	*EGFR*	p.L858R	SNV	45.00	0	0	No
P2	AC	IV	chr7:55242466	*EGFR*	p.746_748del	DEL	39.40	0	0	No
P5	AC	IV	chr12:25398284	*KRAS*	p.G12A	SNV	15.20	0	0	No
P17	AC	IV	chr7:55259524	*EGFR*	p.L861Q	SNV	16.30	0	0	No
P23	AC	IIIA	chr7:55242466	*EGFR*	p.746_750del	DEL	10.50	0	0	No
P25	AC	IV	chr7:55242466	*EGFR*	p.L858R	SNV	15.50	0	0	No
P26	AC	IV	chr2:29445213	*ALK*	p.L1171T	SNV	10.70	0	0	No

AC: adenocarcinoma; SNVs: single nucleotide variations; DEL: deletion, WBC: white blood cell.

**Table 3:** Gene fusions detected in tissue DNA and plasma ctDNA

Patients	Cancer type	Tumor stage	5ʹ gene-exon	3ʹ gene-exon	Fusions	Detected fusion reads		Concordance (Yes or No)	Confirmation
						Tissue DNA	Plasma		
P24	AC	IV	*KIF5B-exon 15*	*RET-exon 12*	KIF5B-RET	77	0	No	Sanger
P26	AC	IV	*EML4-exon 2*	*ALK-exon 19*	EML4-ALK	11	13	Yes	FISH
P28	AC	IV	*EML4-exon 7*	*ALK-exon 19*	EML4-ALK	4	15	Yes	Sanger
P30	AC	IV	*EML4-exon 7*	*ALK-exon 19*	EML4-ALK	214	379	Yes	IHC
P33	AC	IV	*EML4-exon 13*	*ALK-exon 19*	EML4-ALK	93	0	No	NA

AC: adenocarcinoma; NA: not available for validation.

The sensitivity of detecting mutations in plasma ctDNA was 68.5% (18.5/27, 95%CI is 47.8–84.1%) and the positive predictive value (PPV) was 100% (18.5/18.5, 95%CI is 78.6–100%); the specificity was 100% (12/12, 95%CI is 69.9–100%) and the negative predictive value (NPV) was 58.5% (12/20.5, 95%CI is 35.4–78.7%) and the overall concordance between tissue and plasma was 78.21% (30.5/39). The mutation frequencies of SNVs and indels from tissue DNA (average of 24.10%, with a range of 0.30%–52.00%) were higher than that from matched plasma ctDNA (average of 3.70%, with a range of 0.20%−11.60%).

### NSCLC-related driver gene mutations in matched tissue DNA and plasma ctDNA

**Figure 3:**
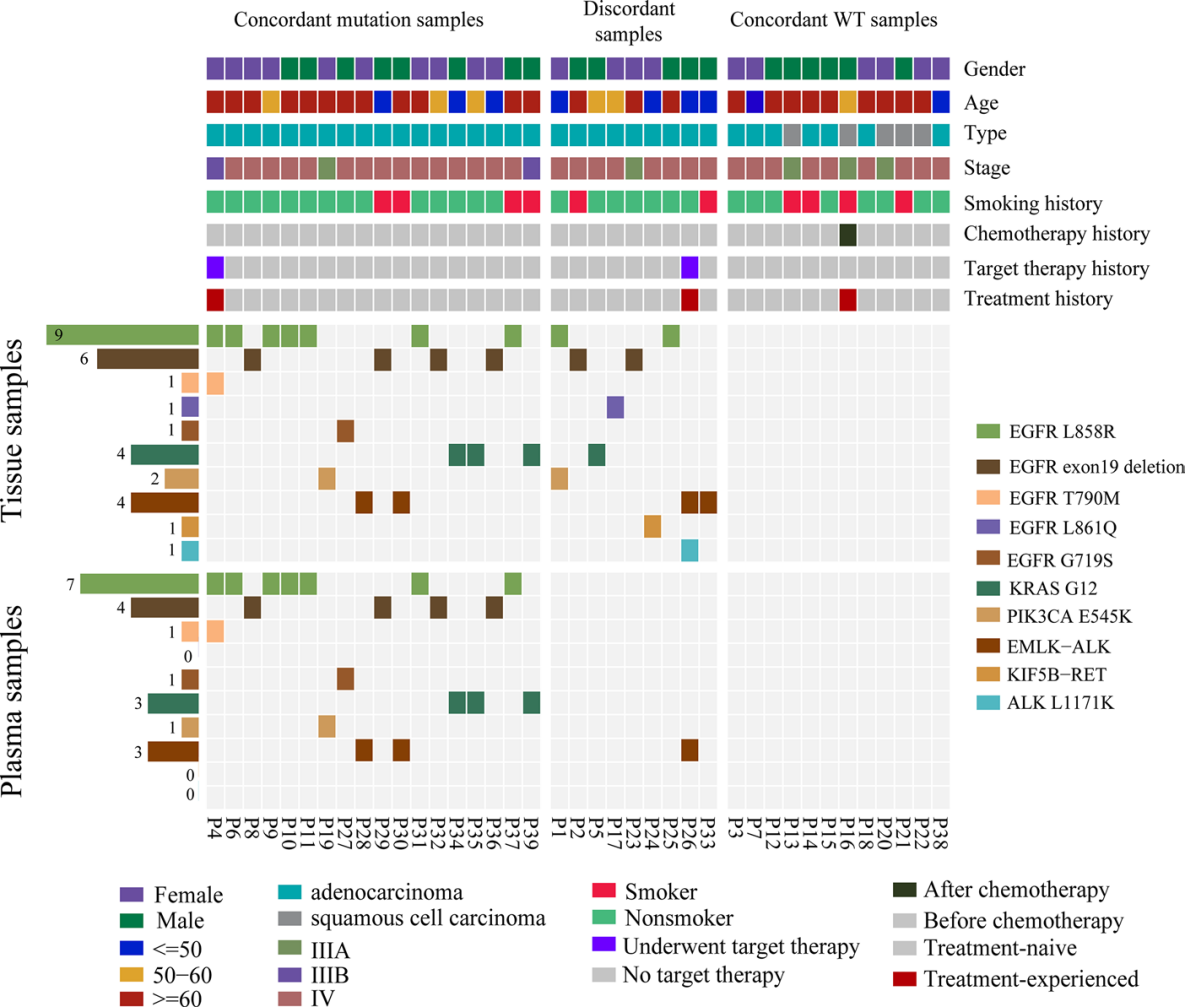
Mutation patterns of tissue and plasma samples from 39 patients with non-small cell lung cancer Clinical characteristics of all 39 NSCLC patients according to the legend. Mutation patterns of tissue and plasma samples from 39 patients are shown in the heat map. Gene mutation frequencies in tissue and plasma samples are shown on the left. All the SNVs and indels detected in discordant samples were only from tissue samples.

Our research priority was to determine the concordance between tissue DNA and plasma ctDNA in NSCLC-related mutation profile, which has high prognostic and therapeutic significance. In the 39 advanced NSCLC patients, we identified gene mutations, including *EGFR*, *KRAS*, *PIK3CA*, *ALK* and *RET* (Figure 3) and the sensitivity, specificity, PPV and NPV of detecting those mutations in ctDNA were illustrated in Table 4. For gene rearrangements, 5 were detected from tissue DNA, and 3 were successfully identified in matched plasma ctDNA (Figure 3). *EML4-ALK* gene fusions were detected in both the tissue and matched plasma of three patients (P-26, P-28 and P-30) and confirmed by routine clinical approaches (FISH, Sanger sequencing or IHC; Table 3). One discordant mutation, *KIF5B-RET* gene fusion in P-24, was only found in tissue DNA, as validated by Sanger sequencing (Table 3). Another inconsistent mutation, *EML4-ALK* gene fusion, was only observed in a tissue sample from patient P-33. We were not able to further verify this gene fusion by Sanger sequencing because the DNA has been run out. But we observed a strong signal (93 reads) in the bam file of the tissue samples (Table 3). Concordant results between NGS and routine clinical approaches (FISH, IHC or Sanger sequencing) demonstrated that target sequencing approach using ctDNA has significant potential in detecting driver gene rearrangements in NSCLC patients.

**Table 4:** The performance of mutation detected in plasma ctDNA

Genes	Sensitivity (%)	Positive predictive value (%)	Specificity (%)	Negative predictive value (%)	Concordance (%)
*EGFR*	70.6 (12/17)	100 (12/12)	100 (22/22)	81.5 (22/27)	84.18 (34/39)
*KRAS*	75 (3/4)	100 (3/3)	100 (35/35)	97.22 (35/36,)	97.44 (38/39)
*PIK3CA*	50 (1/2)	100 (1/1)	100 (37/37)	97.37 (37/38)	97.44 (38/39)
Gene fusions	60 (3/5)	100 (3/3)	100 (34/34)	94.44 (34/35)	94.87 (37/39)
In total	68.5*(18.5/27)	100 (19/19)	100 (12/12)	58.54 (12/20.5)	78.21 (30.5/39)

* : P26 harbored an ALK p.L171T which was only found in tissue DNA and an EMLK-ALK mutation which was found in both tissue DNA and plasma ctDNA. To maintain a total sample number of 39, P26 was counted as 0.5 in each category.

**Figure 4:**
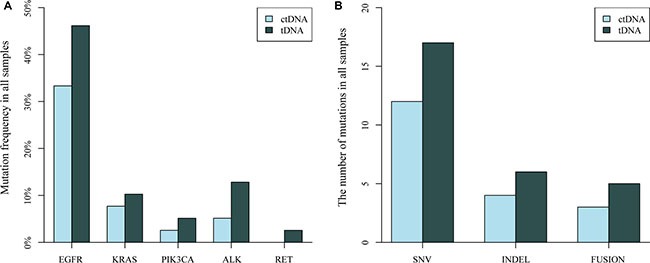
Comparison of the distribution of driver gene mutations identified in NSCLC tissue DNA (tDNA) and plasma DNA (ctDNA) samples (**A**) Mutation frequency of *EGFR*, *KRAS*, *PIK3CA*, *ALK* and *RET* in 39 sample pairs. (**B**) The number of mutation types (SNV, INDEL and gene fusion) detected in 39 paired samples.

In summary, 30 mutations in 39 patients were identified in tissue samples, and the overall mutation percentages of *EGFR*, *KRAS*, *PIK3CA*, *ALK* rearrangements detected from plasma ctDNA were similar with that from tissue DNA (Figure 4A). We detected 17 SNVs, 6 indels, and 5 gene fusions in tissue DNA samples and 12 SNVs, 4 indels and 3 fusions in plasma ctDNA (Figure 4B). There was no significant difference of mutational frequencies for each genes and variant type between plasma ctDNA and tissue DNA.

### Ultra-low frequency mutations analysis

Minimum mutation frequencies detected in matched tissue and plasma samples were 0.30% and 0.20%, respectively (Table 5). All SNVs and indels detected in matched tissue DNA and plasma ctDNA were carefully evaluated using the Integrative Genomics Viewer (IGV). We did not find any reads supporting those variants in the bam files of the matched plasma ctDNA. In addition, digital PCR was used to validate mutations with ultra-low allele frequencies (< 0.50%) and discordant mutations (Table 5). Mutation frequencies detected by NGS were similar to those detected by digital PCR (*R*^2^ = 0.97). In the plasma ctDNA of patients P-1, P-23 and P-25, the mutation frequencies detected by digital PCR were zero or extremely low (less than 0.16%). In the tissue or plasma samples of patients P-4, P-8, P-11 and P-37, the mutations detected by NGS (mutated allele frequency > 0.20%) were successfully detected by digital PCR. These data suggested that our NGS data is agreement with that of digital PCR.

**Table 5:** Ultra-low frequency mutations verified by digital PCR

Patients	Cancer type	Cancer stage	Position	Gene	Mutation	Mutation type	% variants by NGS		% variants by digital PCR	
							Tissue	Plasma	Tissue	Plasma
P1	AC	IV	chr3:178936091	*PIK3CA*	p.E545K	SNV	23.30	0	25.50	0.10
			chr7:55259515	*EGFR*	p.L858R	SNV	45.00	0	40.20	0
P4	AC	IIIB	chr7:55249071	*EGFR*	p.T790M	SNV	0.30	7.70	0.46	6.90
P8	AC	IV	chr7:55242465	*EGFR*	p.745_750del	DEL	20.00	0.20	NA	0.47
P11	AC	IV	chr7:55259515	*EGFR*	p.L858R	SNV	25.00	0.70	25.04	0.93
P23	AC	IIIA	chr7:55242466	*EGFR*	p.746_750del	DEL	10.05	0	17.55	0
P25	AC	IV	chr7:55242466	*EGFR*	p.L858R	SNV	15.50	0	13.65	0.16
P37	AC	IV	chr7:55259515	*EGFR*	p.L858R	SNV	12.70	0.20	NA	0.25

AC: adenocarcinoma; SNVs: single nucleotide variations; DEL: deletion, NA: not available by digital PCR.

## DISCUSSION

In matched tissue and plasma samples obtained from 39 advanced NSCLC Chinese patients, we applied targeted sequencing to detect various NSCLC-related driver gene mutations. In addition to the SNVs and indels, gene rearrangements were also analyzed from both tissue DNA and plasma ctDNA. The overall concordance between tissue and plasma DNA was 78.21% and overall sensitivity and specificity of detecting mutations in ctDNA were 68.5% (95% *CI* = 47.8–84.1%) and 100% (95% *CI* = 69.9%–100.0%), respectively. These results indicated that NGS data regarding driver gene mutations in ctDNA could be useful for molecular diagnostics in advanced NSCLC patients.

In our study, some mutations were only found in tissue DNA but not in plasma ctDNA. This may be due to genomic DNA from necrotic white blood cells is released into the blood and diluted ctDNA in the plasma [21]. The amount of ctDNA in cancer patients likely associated with tumor burden, status of metastasis, vascularity, cellular turnover, and response of therapy [22, 23]. In addition, it is also influenced by clearance, degradation and other physiological filtering events involving blood and lymphatic circulation [21]. However, four ultra-low frequency mutations (minimum mutation frequency of 0.20%) were detected by NGS, which were verified by digital PCR (Table 4), indicating that using plasma ctDNA to detect low frequency somatic mutations in NSCLC patients is applicable. Although the mutation frequency and gene mutation number in plasma ctDNA were less than that in tissue DNA, plasma ctDNA displayed similar driver gene mutation profile with tissue DNA. Therefore it is clinically beneficial to detect gene mutation in plasma ctDNA in advanced NSCLC patients.

Many studies have shown the sensitivity of detecting gene mutation in plasma ctDNA is largely dependent on detection techniques. The sensitivity of *EGFR* mutation detected in ctDNA is 80.3% (49/61) by BEAMing [24], 66.7% (34/51) by PNA-PCR [25], 72.1% (44/61) by ARMS [26]. In our study, using a NGS method of targeted sequencing based on Illumina HiSeq platform the sensitivity of *EGFR* mutation detected in plasma ctDNA was 70.6%. Apparently, the sensitivity of EGFR mutations detection in ctDNA by NGS was not quite different from other methods. However, the greatest advantage of NGS is that it can detect large-scale gene mutations simultaneously whereas others cannot. In addition to the *EGFR* mutations, the sensitivity of detecting *KRAS, PIK3CA* and gene fusions (*EML4-ALK* and *KIF5B-RET*) in plasma ctDNA was 75%, 50% and 60%, respectively. Sacher *et al*. using digital PCR to detect *EGFR* and *KRAS* mutations in plasma samples with sensitivity of 78.4% and 64%, respectively [27]. For several discordant mutations and the mutations with ultra-low frequencies (< 0.50%) by NGS, we also performed digital PCR to validate the data. Our data revealed that our NGS results were highly consistent with digital PCR results.

*EGFR* mutations predict better outcomes in NSCLC patients with EGFR tyrosine kinase inhibitors therapy [28, 29]. In present work, we showed the *EGFR* mutations rate of 43.6% (17 of 39) in tissue DNA and 30.77% (12 of 39) in plasma ctDNA. Previous studies have showed that *EGFR* mutation frequency in tumor is 28.4% (147/517) [30] and plasma samples is 34.3% (79 of 230) [31] in Chinese populations, respectively. As far as we known, sex, smoking status and histology types correlated significantly with EGFR mutation frequency [15] and EGFR mutation are more commonly detected in non-smokers [30]. However, due to the small cohort of patients used in this work, we did not observed a significant difference or correlation between the driver gene mutations or *EGFR* mutations and smoking history or disease stages (data not shown). The small discrepancies in *EGFR* frequency between our results and previous studies may mainly arise from the small cohort of patients. Additionally, the incidence of *KRAS*, *PIK3CA* and *EML4-ALK* mutations in the present work were 10.3%, 5.1%, 12.8% in tissue DNA and 7.7%, 2.6% and 7.7% in plasma ctDNA which were consistent with previous reports [32].

Since acquired resistance is very common in NSCLC patients after treated with target therapy for a period, assessment the driver gene mutations profile is essential for diagnostics and target therapy for NSCLC patients. Patients with EGFR L858R were sensitive to EGFR-TKIs, such as gefitinib [33] and erlotinib [34], but almost half of the patients developed resistance to EGFR-TKIs by acquiring the secondary mutation T790M [35, 36]. Patient P-4 was first identified *EGFR* exon 21 mutation by liquid biochips. After first line treatment with gefitinib for one and a half years, the patient appeared to acquire EGFR-TKI resistance. At this point, *EGFR* L858R and T790M mutations were found in both tissue and plasma samples by NGS, which could well explain the EGFR-TKI resistance developed in patient P-4. Approximately 3–7% of lung cancer patients harbor *ALK* fusions [37]. Crizotinib treatment was administered to patient P-26 when he was diagnosed with *EML4-ALK* fusion by FISH one and a half years ago. A secondary mutation, ALK L1171T was found in tissue DNA by NGS, which is interpretable for crizotinib resistance in patient P-26 [38]. Clinically, in the vast majority of cases, *ALK* rearrangements are non-overlapping with *EGFR* mutations [39, 40]. However, some evidence showed that the presence of *EML4-ALK* gene rearrangement is associated with EGFR-TKIs resistance among patients with metastatic diseases [41, 42]. In our present work, we did not found ALK rearrangements overlapping with *EGFR, PIKC3A* or *KRAS* in any patients with advanced NSCLC.

The sensitivity of gene fusions detected in matched tissue and plasma samples were less than SNVs, which may be due to the small cohort of patients. There are only 5 gene fusions but 17 SNVs detected in 39 NSCLC patients, which may influence the sensitivity calculation. In fact, the overall concordance of gene fusions in 94.87% (37/39) is higher than SNVs (87.2%, 34/39). It is worthy to notice that the different principle of variant calling approaches. For SNVs, we used minimum number of reads carrying the mutation to find the real mutations, but for gene fusions we used the minimum number of soft-clip reads to obtain the true fusions. The filters for gene fusions are more stringent than SNVs and that's why the low frequency of gene fusions was not found in plasma samples.

In conclusion, the sequencing approach described herein can be used to detect gene mutations, such as SNVs, indels and gene fusion, in both plasma and tumor tissue from patients with advanced NSCLC. Our results showed a high concordance of gene mutations found in plasma samples and paired tissue samples. We therefore believe that ctDNA in plasma is likely to become an alternative source for cancer-related mutations profiling in advanced NSCLC patients, and is useful for molecular diagnostics, prognosis and targeted drug selection.

## MATERIALS AND METHODS

### Ethics statement

This study was approved by the Ethics Committees of The First Affiliated Hospital of Xi’an Jiao Tong University and Tianjin Medical University General Hospital. Thirty-nine patients with advanced NSCLC were recruited and patients signed informed consent for use of their blood plasma and tissue biopsy. All clinical data and samples were received anonymously.

### Sample collection and DNA extraction

Peripheral blood was collected before surgery in one week for all the patients. Tissue samples were collected from the metastatic nodules for 11 patients, who were found pleura metastasis in the surgery. For the others without pleura metastasis, tissue samples were collected from primary site. Three sample types were examined for mutation profiles: fresh or formalin-fixed paraffin embedded (FFPE) tumor tissue, peripheral blood lymphocytes and plasma. The QIAAmp nucleic acid kit (Qiagen NV, Venlo, The Netherlands) and QIAAmp DNA FFPE tissue kit (Qiagen) were used in DNA extraction from fresh frozen biopsy and FFPE samples, respectively. Peripheral blood samples were collected in cfDNA BCT tubes (Streck Laboratories, Omaha, NE), stored at 15–30°C and processed within 72 hours. Each tube was centrifuged at 1600 *g* for 10 minutes at room temperature. Pellets containing peripheral blood lymphocytes were stored at −20°C for further use. Aliquots of plasma were centrifuged at a maximum speed at 16000 *g* for another 10 minutes. The supernatant was transferred to sterile 1.5 ml tubes and stored at −80°C before extraction. Circulating tumor DNA was extracted from 5 ml plasma with the QIAamp circulating nucleic acid kit (Qiagen) following the manufacturer's instructions using a QIAvac 24 plus vacuum manifold (Qiagen). To enhance ctDNA yields, carrier RNA was added to lysis buffer. Germline genomic DNA from peripheral blood lymphocytes was extracted using the RelaxGene Blood DNA System (TianGen Biotech Co., Ltd., Beijing, China). DNA from both tissue biopsy and plasma samples were quantified by Qubit 2.0 (Life Technologies) according the recommended protocol.

### Library construction, hybridization and sequencing

The library was constructed using a KAPA Hyper Prep kit (Kapa Biosystems) according to the manufacturer's instructions. The prepped libraries were hybridized with two different hybridization reagents and blocking agents in SureSelectXT and SureSelectQXT Target Enrichment System (Agilent Technologies). Because we used adaptors which were different from those included in the kits, additional blocking oligos and a P5/P7 primer were applied instead of the primers provided. The size of the prepped library was qualified using a 2100 Bioanalyzer (Agilent Technologies), and quality was assessed using the StepOnePlus real-time PCR system (Life Technologies). The concentration of each library was quantified using a QPCR NGS library quantification kit (Agilent Technologies). Multiplexed libraries were sequenced using a HiSeq platform (Illumina, San Diego, CA).

A panel covering 40 cancer-related genes, such as *EGFR*, *BRAF*, *KRAS*, *PIK3CA*, *ALK*, *RET* and *ROS1*, was used in this study. All genes, including oncogenes and tumor suppressor genes, were evaluating using full exon tiling arrays. For *ALK*, *ROS1* and *RET*, introns where rearrangements usually occur were also included.

### Variant calling

Genomic alterations, such as point mutations, indels and gene rearrangements, were assessed in tissue samples, white blood cells and the plasma of all patients. Pre-alignment quality control was performed for each sample. Clean reads were obtained after removing adaptor sequences and low mapping quality reads. Reads aligned to reference genome hg19 were performed with BWA [43]. Duplication reads were marked and removed by picard. Variants in white blood cells were used to filter germline mutations. Somatic mutations were determined using the following filters: (i) the minimum average sequencing depth of the target for tissue samples was at least 200 × and at least 1000 × for plasma DNA; (ii) the minimum number of reads carrying the mutation was > = 5; and (iii) variant allele frequency > = 0.2%. Every somatic mutation identified in tDNA and ctDNA was checked by Integrative Genomics Viewer (IGV) software [44] and Samtools software. For point mutations and indels, variant frequencies less than 0.5% were further verified by the QuantStudio 3D digital PCR system (Life Technologies).

### Statistical analysis

For statistical analysis, tissue DNA was considered a reference in considering concordance between tissue and plasma DNA. Matched tissue and plasma samples carrying the same gene mutations were considered true positives (TP). Matched tissue and plasma samples without somatic mutations were true negatives (TN). Gene mutations found in tissue samples, but not plasma samples, were classified as false negatives (FN), and gene mutations found in plasma samples, but not tissue samples, were false positives (FP). One sample with both false negative and positive mutation were counted as 0.5 in each category to maintain a total sample number of 39. Sensitivity, specificity and concordance were calculated according to TP, TN, FN and FP. The relationship between sample characteristics and gene mutations was measured by Fisher's exact or chi-square tests as appropriate. All statistical analyses were performed by R3.2.2.
